# Detection of Aspergilloma Disease Using Feature-Selection-Based Vision Transformers

**DOI:** 10.3390/diagnostics15010026

**Published:** 2024-12-26

**Authors:** Siyami Aydın, Mehmet Ağar, Muharrem Çakmak, Mustafa Koç, Mesut Toğaçar

**Affiliations:** 1 Department of Thoracic Surgery, Faculty of Medicine, Firat University, 23119 Elazig, Turkey; md.mehmetagar@gmail.com (M.A.); g.c.dr.ckmk@gmail.com (M.Ç.); 2 Department of Radiology, Faculty of Medicine, Firat University, 23119 Elazig, Turkey; mkoc44@yahoo.com; 3 Department of Management Information Systems, Faculty of Economics and Administrative Sciences, Firat University, 23119 Elazig, Turkey; mtogacar@firat.edu.tr

**Keywords:** aspergilloma disease, aspergilloma detection, vision transformers, merge-based feature selection, machine learning

## Abstract

**Background**: Aspergilloma disease is a fungal mass found in organs such as the sinuses and lungs, caused by the fungus *Aspergillus*. This disease occurs due to the accumulation of mucus, inflamed cells, and altered blood elements. Various surgical methods are used in clinical settings for the treatment of aspergilloma disease. Expert opinion is crucial for the diagnosis of the disease. Recent advancements in next-generation technologies have made them crucial for disease detection. Deep-learning models, which benefit from continuous technological advancements, are already integrated into current early diagnosis systems. **Methods**: This study is distinguished by the use of vision transformers (ViTs) rather than traditional deep-learning models. The data used in this study were obtained from patients treated at the Department of Thoracic Surgery at Fırat University. The dataset consists of two class types: aspergilloma disease images and non-aspergilloma disease images. The proposed approach consists of pre-processing, model training, feature extraction, efficient feature selection, feature fusion, and classification processes. In the pre-processing step, unnecessary regions of the images were cropped and data augmentation techniques were applied for model training. Three types of ViT models (vit_base_patch16, vit_large_patch16, and vit_base_resnet50) were used for model training. The feature sets obtained from training the models were merged, and the combined feature set was processed using feature selection methods (*Chi*2, mRMR, and Relief). Efficient features selected by these methods (*Chi*2 and mRMR, *Chi*2 and Relief, and mRMR and Relief) were combined in certain proportions to obtain more effective feature sets. Machine-learning methods were used in the classification process. **Results**: The most successful result in the detection of aspergilloma disease was achieved using Support Vector Machines (SVMs). The SVM method achieved a 99.70% overall accuracy with the cross-validation technique in classification. **Conclusions**: These results highlight the benefits of the suggested method for identifying aspergilloma.

## 1. Introduction

*Saprophytic Aspergillus* species are filamentous fungi that thrive in a moist environment and are found in soil, plants, and ubiquitously. *Aspergillus* fumigatus is the most common human pathogen, but A niger, A flavus, and A oxyzae have also been reported to cause human disease. Aspergillomas colonize a poorly drained, avascular cavitary space, adhere to the wall with their conidia and bud, and cause an inflammatory response [1]. Aspergilloma is common in immunocompetent patients with structural lung disease. It is particularly common in patients with tuberculosis. Approximately 10% of tuberculosis-related lung cavities develop aspergilloma [2]. Aspergilloma can present with a variety of clinical symptoms ranging from asymptomatic to fatal hemoptysis. Hemoptysis is the most common clinical symptom, occurring in 54–87.5% of cases according to various case series [3]. Fever is rare, but symptoms such as cough, chest pain, malaise, and weight loss may be present. The diagnosis and treatment of aspergilloma is complex and difficult. This is because the radiographic features are similar to those of other lung diseases [4].

The diagnosis of aspergilloma is based on radiographic features in patients with underlying lung disease. In addition to radiographs, bronchoalveolar lavage (BAL) cultures, positive serum or BAL galactomannan tests, and serum *Aspergillus*-specific IgG antibodies are used in the diagnosis. Surgical resection is the gold standard treatment for aspergilloma. The efficacy of antifungal treatment is limited [5]. Conservative treatment is recommended in asymptomatic patients with a single stable aspergilloma, and, if symptoms develop, resection without contraindication is recommended. Long-term triazole courses are recommended for those at high surgical risk.

A few artificial intelligence (AI)-based studies of aspergilloma disease have recently appeared in the literature. Some of these studies are listed in Table 1.

Tong Liu et al. [6] used a deep-learning model capable of differentiating between Staphylococcus aureus pneumonia and *Aspergillus* pneumonia in their study. They trained the model using computed tomography (CT) images of 60 patients and used a pre-trained model, DenseNet-201. After training, the model achieved an overall accuracy of 84.3%. Wei Wang et al. [7] designed a deep-learning model trained on large datasets (pre-trained) in their study. This model, named IPA-NET, underwent transfer learning using 300,000 CT images. They achieved an accuracy of 96.8% in their prototype model for the early diagnosis of invasive *Pulmonary aspergillosis*, based on training with large-scale data. L.N. Walti et al. [8] utilized machine-learning methods to assess the probability of developing aspergillosis in lung transplant recipients. They compared the postoperative and one-year status to detect *Aspergillus* colonization in relevant patients. They used the naive Bayes, decision tree, and simple logistic regression methods for accurate classification. They achieved the best classification result with a 91.7% accuracy using the decision tree method.

There is limited literature on AI-based studies related to the diagnosis of aspergilloma. Studies in the literature have traditionally used deep-learning models and machine-learning methods, which are considered classical. Wei Wang et al. [7] achieved successful model training due to an adequate number of datasets. However, if they had performed analysis using pre-processing techniques such as data augmentation, data normalization, region of interest cropping, etc., and post-processing techniques such as feature extraction, fusion, etc., they may have achieved better performance. Our proposed model includes all these processing steps (pre-processing and post-processing) and uses next-generation transformers for analysis instead of classical deep-learning models.

This study proposes a hybrid artificial intelligence approach for the effective detection of aspergilloma disease. In the hybrid approach, pre-processing steps were applied to facilitate model training. Vision Transformer (ViT) models were used to train the data. Features extracted from the final layers of the ViT models were processed using feature selection algorithms to extract more efficient features. Efficient features were processed using a merging technique and classified using machine-learning methods. All these steps are expected to contribute positively to the aspergilloma classification process. The innovative aspects and contributions of the proposed approach are as follows:

- Pre-processing steps used to crop unnecessary regions from the input images, and data augmentation techniques used to facilitate the training of ViT models;
- The preference for transformers, which are next-generation technologies that have recently been shown to outperform traditional deep-learning models, when training models and extracting efficient features from them;
- The reduction in the dimensionality of feature sets extracted from transformers using feature selection algorithms (cost savings), and the detection of aspergilloma disease using a merging technique with machine-learning methods (performance improvement);
- The incorporation of AI-based approaches to assist physicians and radiologists in the diagnosis of aspergilloma disease.

The remaining sections of the article are organized as follows: Section 2 provides details on the dataset, techniques, methods, and models used in this study. Section 3 explains the proposed feature selection-based ViT hybrid approach. Section 4 presents the experimental analysis. The last two sections of the paper include the discussion and conclusion, respectively.

## 2. Materials and Approaches

This section consists of subsections providing information about the dataset, techniques, methods, and models. Detailed information is provided about the data of patients with aspergilloma obtained from Fırat University Hospital (Elazig, Turkey). In addition, the approaches used in the proposed hybrid model are detailed in this section. The preferred hyperparameters and their corresponding values for each approach are provided. For hyperparameters not explicitly mentioned in the paper, the default values were used for the analysis.

### 2.1. Dataset

**Figure 1:**
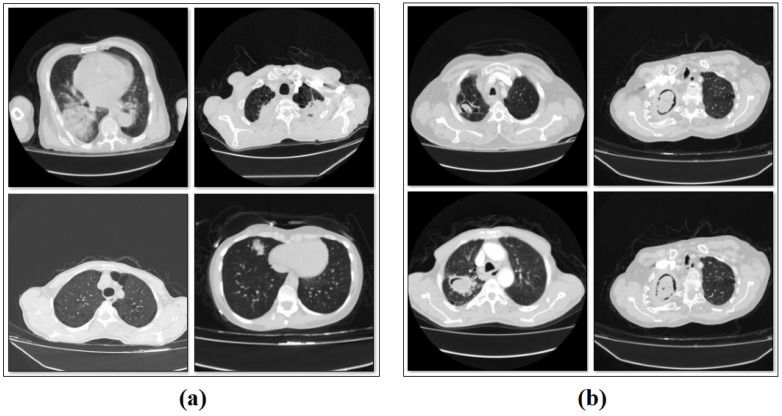
Sample images from the classes/types of the dataset: (**a**) patients without aspergilloma, and (**b**) patients with aspergilloma.

In this retrospective study, a total of 168 patients with pathologically diagnosed aspergilloma and diseases confused with aspergilloma between 2011 and 2024 were included. The images of the dataset were obtained from the department of Fırat University Research Hospital (Elazig, Turkey). Eighty-four of the patients were pathologically diagnosed with aspergilloma, and the other 84 patients had diseases confused with aspergilloma. In the chest CT scans of the patients in both groups, two chest CT images of the disease were obtained, and a total of 336 slices were obtained. There were 48 male and 36 female patients in the aspergilloma group and 52 male and 32 female patients in the non-aspergilloma group. The mean age of the aspergilloma group was 64.6 ± 13.8 years and the mean age of the non-aspergilloma group was 60.3 ± 16.8 years. The other class of the dataset included 168 CT images without aspergilloma disease. Thus, the dataset consists of 168 images with aspergilloma and 168 images without aspergilloma. The classes are balanced in number. The total number of original images is 336. An example subset of images related to the types of the dataset is shown in Figure 1.

### 2.2. Proposed Hybrid Approach

The proposed approach consists of AI-based methods and models that can assist in diagnosing aspergilloma disease by analyzing CT images. To achieve the objective of this study, ViT models, which have recently played a more effective role in image processing and efficient feature extraction than Convolutional Neural Network (CNN) models, have taken the main role. In order to add a more innovative aspect to these models and to increase the diagnostic success of the disease, we synthesize the model with hybrid approaches. The proposed hybrid model consists of three main steps in terms of design. These steps are pre-processing, model training and feature extraction, and post-processing step.

In the pre-processing step, image enhancement and data augmentation processes are performed. Raw data acquired in a hospital environment may have different resolutions. In the pre-processing step, each variable-resolution image is cropped to images with a fixed resolution of 224 × 224. The reason for choosing 224 × 224 resolution is that ViT models process the image at this resolution. Then, due to the limited number of datasets, a data augmentation technique was used. ViT models (basic patch16-224, large patch16-224, and base resnet50-224-in21k) are used in the model-training step. The reason for the variety of ViT models is to enrich the feature sets without relying on a single model. During model training, the data augmentation technique is applied only to the training data (not the test data). This prevents situations like overfitting. The feature sets of the images are extracted before the last layer (linear layer), where the classification process of the three ViT models takes place. In the feature extraction step, the “base”-based ViT models (base patch16-224, and base resnet50-224-in21k) provide 768 features, while the “large”-based model (large patch16-224) provides 1024 features. Then, the features obtained from the three ViT models (768 + 768 + 1024) are combined to obtain a new combined feature set containing 2560 features. The goal here is to enrich the number of features so that the most efficient features can be selected for the next feature selection.

**Figure 2:**
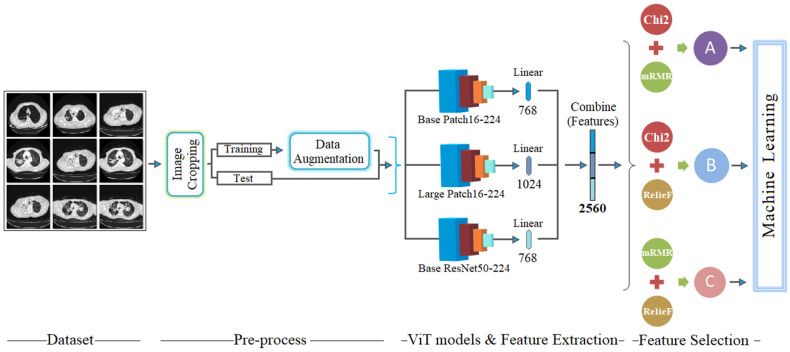
General design of the proposed hybrid model.

In the final processing step, the merged feature set (2560 features) is processed with *Chi*2, mRMR, and Relief techniques, respectively, to select the efficient features. These feature selection methods rank each of the 2560 features with specific scores according to their mathematical operations. A table is created in which the features are ranked from the highest score to the lowest score. From the ranked features selected by the feature selection methods, we select a certain number of features (such as the best 100 features) and perform the classification process using machine-learning methods (kNN, LDA, and SVM). The advantage of this process is the ability to achieve the same performance with sets containing a large number of features as with sets containing a small number of features. Then, taking into account the ranked features obtained with each feature set method, the best feature sets (such as 25 features, 40 features, etc.) are combined and classified with the machine-learning method. Here, feature selection methods (*Chi*2 and mRMR, *Chi*2 and Relief, and mRMR and Relief) are combined with the specified number of features and their performance is measured. Thus, a successful result is obtained with even fewer number of features. The design of the proposed hybrid model is shown in Figure 2.

### 2.3. Pre-Processing Step

Pre-processing steps typically consist of data normalization, data cleaning, data merging, data augmentation, removal of redundant regions, or regional focusing. These choices may vary depending on the type of dataset, size, data volume, or analysis choices. Since the dataset images in this study are of variable resolution and do not consist of a large number of data, we focused on two techniques: data cropping and data augmentation.

For variable-resolution images, the data-cropping technique removes regions that are deemed unnecessary and emphasizes the area of the image that should be in focus. In this study, due to the variability of the input images, we resized them to 256 × 256. Then, we obtained cropped images with a resolution of 224 × 224 from the center of each image. All these operations were performed using the Python-language Torch and Numpy libraries. The reason for choosing the 224 × 224 image resolution is that it is the same as the input resolution of the transformer models. The limited dataset would have limited the training of the transformers. For this study, the data were subjected to horizontal rotation and value normalization using the Python 4.0 software language, but this process (data augmentation) was applied only for the training data.

### 2.4. Machine-Learning Methods for Classification

Linear discriminant analysis (LDA) is a statistically based machine-learning method based on the Fisher method developed by Ronald Fisher and named after him. Many methods are used for classification and data dimensionality reduction. One of the most popular methods is LDA. LDA calculates the classification based on the probability of interpretation. In other words, it is based on finding a linear combination of variables to best distinguish the class types. For that purpose, Equation (1) through (2) apply, with Equation (2) being a score function. The linear coefficients that maximize the score function are estimated using Equations (3) and (4). In these equations, C is the covariance matrix and μ is the mean vector. Z is the output of the model (dependent variable). β represents the linear model coefficient, X represents the data vector, and P represents the class probabilities. $C_{1}$ and $C_{2}$ refer to the coefficients or values of the two groups. $n_{1}$ and $n_{2}$ refer to the sizes (sample numbers) of the two different groups or datasets. These numbers determine the contribution of each group [9,10].

(1) $$Z=\beta_{1}x_{1}+\beta_{1}x_{1}+\ldots +\beta_{d}x_{d}$$

(2) $$S\left(\beta \right)=\frac{\beta^{T}\mu_{1}-\beta^{T}\mu_{2}}{\beta^{T}C\beta }$$

(3) $$\beta =C^{-1}(\mu_{1}-\mu_{2})$$

(4) $$C=\frac{1}{\left(n_{1}+n_{2}\right)}(n_{1}C_{1}+n_{2}C_{2})$$

After these steps, the Mahalanobis equation is used to calculate the best separation between the two groups (Equation (5)). When examining the variables in Equations (5) and (6), Δ represents the Mahalanobis difference between the two groups, X represents the data vector, and P represents the class probability values. If the condition in Equation (6) is satisfied, a new feature is classified.

(5) $$\Delta^{2}=\beta^{T}(\mu_{1}-\mu_{2})$$

(6) $$\beta^{T}\left(x-\left(\frac{\mu_{1}+\mu_{2}}{2}\right)\right)>\log\;\frac{P{(C}_{1})}{P{(C}_{2})}$$

The Support Vector Machine (SVM) is a machine-learning method commonly used for both classification and regression. SVM processes the input data and places the resulting features on an abstract coordinate plane. The data in the coordinate plane form boundaries in binary or multiple ways. Thus, they form the hyperplane region. The maximum boundary area is determined by optimization. SVM processes the features in the hyperplane and classifies them. Equation (7) is used for this processing step. Here, $x_{i}$ and $y_{i}$ represent the coordinate points of the features in the hyperplane. The variable w represents the edge width and b represents the bias [11,12].

(7) $$y_{i}(\vec{w}\cdot {\vec{x}}_{i}-b)\geq 1,\;\forall i$$

The k-nearest neighbor (kNN) method is a supervised machine-learning method commonly used in classification. The algorithmic structure of the method takes into account the initially defined data point and uses the nearest neighbor data around the data point. The number of nearest neighbor data are represented by the parameter k, and the query is based on the closest distances. In the next step, the k-nearest data points are found and voting is performed. In the final case, the data points in the class with more votes are displayed. This is repeated within a given iteration [13,14].

The important parameters and values of the preferred machine-learning methods for the proposed hybrid model are given in Table 2. These values are the default values of the methods. Machine-learning methods for classification are implemented using MATLAB 2024a, based on the default values of the hyperparameters.

### 2.5. Feature Selection Methods

In AI-based models or approaches, feature selection methods are used to increase classification success. In this way, the goal is to increase the success by highlighting the most efficient features from the feature set obtained from the model layer. In short, such methods allow us to create a new sub-feature set by selecting better features from the existing feature set [15,16]. Three types of feature selection methods were used in this study.

#### 2.5.1. *Chi*2 Method

The *Chi*2 is a statistically based hypothesis method that is preferred for analyzing contingency tables when the dimensions of the feature sets are sufficiently large. It is generally more effective for datasets with two-category features. For smaller feature sets, the Fisher method is generally preferred [17]. The *Chi*2 formula used in feature selection methods is as shown in Equation (8):

(8) $$Chi2=\sum_{i=1}^{n}{\left(O_{i}-E_{i}\right)}^{2}/E_{i}$$

where the variable “$O_{i}$” represents the observed number in each category and the variable “$E_{i}$” represents the expected number in the category represented by “$O_{i}$”. The *Chi*2 method is a test of the significance of the difference between the expected and observed raw frequencies. It is also often used in feature selection methods and generally works better when evaluating raw frequencies that contain dichotomous features [17].

#### 2.5.2. mRMR Method

The Minimum Redundancy Maximum Relevance (mRMR) algorithm is a filtering method for selecting the most appropriate features by processing the feature sets extracted from the input data. It gives successful results in binary classes. A number representing the features is given in order and it tries to establish a relationship between the feature numbers using its own algorithm. It also determines the relationship link with a score. The one with a higher score is judged to have a stronger association. Thus, the mRMR method determines the weakest features as residual features and the strongest features as suitable (relevance) features. In short, it looks for similarity between features [18,19].

Here, the maximum relevance (W) and minimum redundancy (V) values are calculated by the mRMR algorithm. V usually represents the relationship of the features to the target variable. This gives a measure of the mutual information of each feature with the target variable and implies maximum relevance. W measures the similarity or correlation between selected features. This can increase the complexity of the model or risk overfitting. Therefore, V means that unnecessary similarities between the selected features should be minimized. Therefore, Equation (9) combines the search for high relevance and low redundancy, and the max function sets a goal to select the features that maximize this ratio. Finally, a score table is generated according to Equation (9) and the score of each feature is calculated. And a ranking among the feature scores is carried out.

(9) $$Maximize:\;(\frac{V}{W})$$

#### 2.5.3. Relief Method

The Relief method helps determine the most efficient features in a high-dimensional feature space by using an approach similar to nearest neighbor calculations between features. In the Relief method, the number of nearest neighbors is determined by a random algorithm when selecting features, and the random feature is also used to determine the thresholds. This may have advantages or disadvantages (performance cost, etc.). The Relief method is similar to a univariate method and ranks each attribute separately. It then calculates the interdependencies between all the ranked attributes separately, taking a multivariate approach. This results in a more objective evaluation. For example, in the set of attributes, the nearest neighbors are calculated for each attribute. It also calculates a hyperdimensional decision boundary for each attribute. The goal is to find the target attribute. The score of the target attribute is updated with the neighboring attribute values close to this decision boundary. This makes it easier for Relief to calculate similarities and dissimilarities [20,21].

### 2.6. Vision Transformers

**Figure 3:**
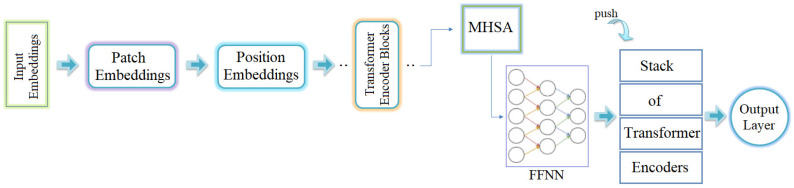
Process stage of the ViT model.

Recent advancements in deep-learning models have significantly contributed to various fields of research. CNNs have made positive contributions to the classification process in autonomous medical image analysis applications. The main drawback of CNN models is that they have not been effective in learning long-range information due to localized receptive fields that limit their capabilities in vision-based processing. ViT models are more effective at transferring long-range information. This is because the concept of attention in image converters works better in ViT models. ViT models are simpler than CNN models in terms of parameter density. However, they can perform better [22,23,24]. The architecture of transformer model consists of six stages [24,25,26]. These process stages are shown in Figure 3.

- The first stage is the input embeddings: The input embeddings receive image data. The images are processed into patches. Each image is first divided into a series of 2-dimensional patches. Each patch can have a specific resolution (16 × 16, 24 × 24, etc.).
- The second stage is patch embedding. In this stage, each patch is processed by an embedding layer. In the last step of this stage, each patch is represented as a vector.
- The third stage is position embedding. In this stage, the order of the patches is determined and position embeddings are added. In this way, the position of each patch can be easily found by the model.
- Transformer encoder blocks are located in stage 4. There is more than one of these blocks in the ViT architecture, each transformer encoder block. It consists of two components: The first component is the Multi-Head Self-Attention (MHSA). The role of this component is to look for correlations between image patches and to model the correlations. The second component is a Feed-Forward Neural Network (FFNN). This component extracts and processes features from each patch and then provides the necessary transformations for the next stage.
- The fifth stage is the transformer encoder stack. This is where the stacking process takes place. This allows deeper features to be extracted and complex correlations to be learned.
- The last stage, as in any model, is the output layer. The data processed by the transformer encoder stack are collected in this layer (output layer). This layer is often used for classification tasks as the last layer of CNN models. If classification is to be performed, activation functions (such as softmax) are preferred in ViT models [22,27].

Three variants of the ViT model were selected for this study. These models are as follows:

- ViT base patch16-224 model: The model accepts images with an input resolution of 224 × 224 and performs operations by dividing each patch image to have a resolution of 16 × 16 pixels. It is a simpler version than the ViT large model. It contains fewer transformer encoder blocks and usually has fewer parameters. It also performs tasks with lower memory consumption, which means it requires fewer computing resources to process the data.
- ViT large patch16-224 model: The model accepts images with an input resolution of 224 × 224 and performs operations by dividing each patch image to have a resolution of 16 × 16 pixels. This model data type is a ViT model specifically designed for larger and higher resolution images. Its architecture has a more complex parameter structure (compared to the other ViT models in this study).
- ViT base resnet50-224-in21k: This model has a hybrid architecture and is a combination of ViT and ResNet architectures. This model uses several convolutional layers from the ResNet-50 architecture, and then the ViT architecture comes into play. The purpose of this design is to take advantage of ViT’s attention mechanism and long-range connections, while taking advantage of ResNet’s deep feature extraction and pattern recognition. It is more preferred in data diversity. It performs its operations by converting the input images to 224 × 224 resolution [26,28,29].

## 3. Experimental Results

The tools used in the proposed approach are as follows: Python 4.0, through the Jupyter Notebook interface, is employed for the pre-processing steps and training transformer models; and MATLAB 2024 is utilized for the feature selection methods and machine-learning methods. The computational resources used for the analysis include an Intel Core i7 processor clocked at 3.40 GHz, with 32 GB of RAM and a graphics card with 10 GB of memory. The ViT models were analyzed using Google Colab servers.

The confusion matrix is generally preferred in classification processes [30]. The confusion matrix was used to validate the analyses of this study. The equations used to calculate the metrics of the confusion matrix are given in Equations (10)–(14) [31,32,33]. When the equations are examined, the results were as follows: positive (*P*), negative (*N*), true (*T*), and false (*F*). In addition, the metric abbreviations used in the equations are as follows: accuracy (*Acc*), f-score (*F-Scr*), specificity (*Sp*), sensitivity (*Se*), and precision (*Pre*). The *Acc* metric is often used in measurement analysis and is effective in balanced datasets. The *F-Scr* metric is considered when there is an unbalanced distribution of class types [34,35].

(10) $$\mathrm{Acc}=\frac{TP+TN}{TP+TN+FP+FN}$$

(11) $$\mathrm{F-Scr}=\frac{2\times TP}{2\times TP+FP+FN}$$

(12) $$\mathrm{Sp}=\frac{TN}{TN+FP}$$

(13) $$\mathrm{Se}=\frac{TP}{TP+FN}$$

(14) $$\mathrm{Pre}=\frac{TP}{TP+FP}$$

The preferred parameters and their values for the ViT models used in this study are shown in Table 3. In the ViT models, except for the parameters in Table 3, other parameter choices are the default values of the models. The experimental analysis processes that took place in deciding on the proposed hybrid approach consisted of five steps. The experimental analysis steps are as follows.

**Figure 4:**
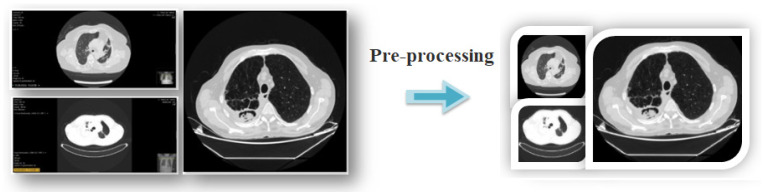
Example illustration of the pre-processing step performed for each image in the dataset.

The first step is to improve the dataset to make it more useful before training the ViT models. For each image, the Python code “ResizedCrop (224)” was used to reconstruct the resolution from the center of the image to 224 × 224. After separating the dataset as training data for ViT models, all the training data were processed using the data augmentation technique. The data augmentation technique was not applied to the test data in model training. The data augmentation technique is generally preferred for datasets with a limited number of images. We used this technique due to the limited size of the dataset. An example design for the pre-processing step performed in the first step is shown in Figure 4.

**Figure 5:**
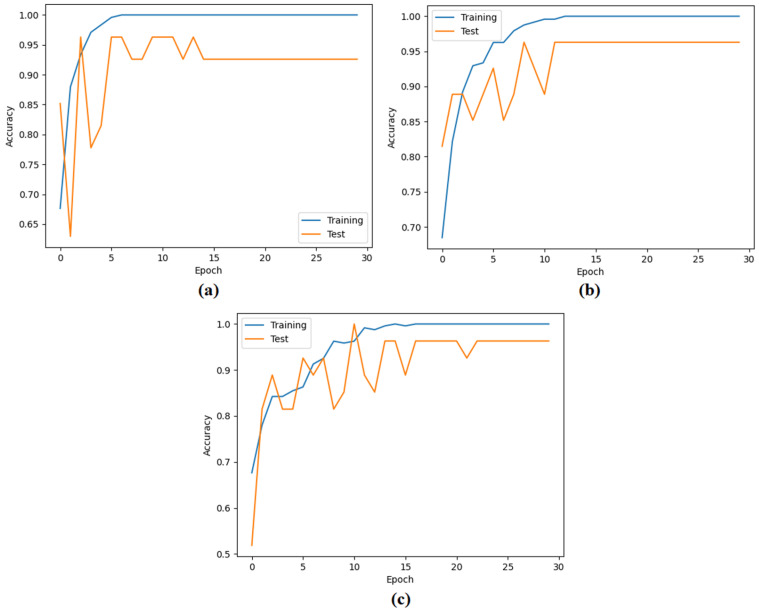
Classification performances of ViT models: (**a**) ViT base patch16-224 model, (**b**) ViT large patch16-224 model, and (**c**) ViT base resnet50-224-in21k.

**Figure 6:**
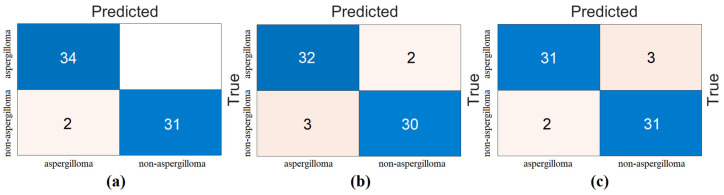
Confusion matrices obtained in the classification process of ViT models: (**a**) ViT base patch16-224 model, (**b**) ViT large patch16-224 model, and (**c**) ViT base resnet50-224-in21k.

In the second step, the data processed in the pre-processing step were each trained with ViT models. The goal of the second step was to extract the feature set obtained in the final layer of each ViT model. In this process, the classification performance of the ViT models was also measured. In the experimental analysis, 80% of the dataset was divided into training data and 20% into test data. The “linear” layer was used in the classification process of the ViT models. The overall accuracy performance graphs of the models are shown in Figure 5. The confusion matrices of the models are shown in Figure 6, and the metric results obtained from the confusion matrices are given in Table 4. Analyzing Table 4, the “Base patch16-224” ViT model gave the best performance and 97.01% overall accuracy was obtained from this model. Other metric results of the ‘Base patch16-224’ ViT model in the detection of aspergilloma class are as follows: the ‘*Se*’ metric success was 100%, ‘*Sp*’ metric success was 93.94%, ‘*Pre*’ metric success was 94.44%, and F-score metric success was 97.14%. Other ViT models also had overall accuracies greater than 90%. It was observed that the ViT models performed successfully in the detection of aspergilloma disease.

**Figure 7:**
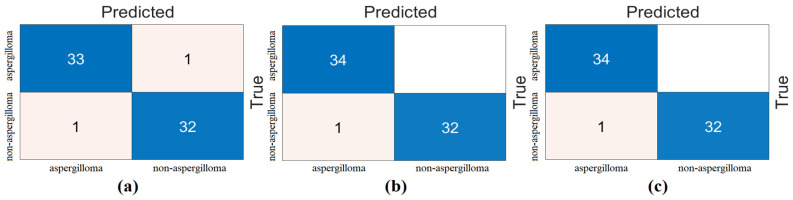
Confusion matrices obtained by processing the combined feature set (2560 features) with machine-learning methods: (**a**) kNN, (**b**) LDA, and (**c**) SVM.

The goal of the second step of the experimental analyses was to combine the ViT models by extracting feature sets from the last layer that performs classification before the “linear layer”. Thus, the feature sets of the three ViT models will be combined to create a new feature set and reclassified with machine-learning methods (kNN, LDA, and SVM). As a result, it would be determined whether the merging technique contributed to the classification success. We found that 768 features from the “Base patch16-224” model, 1024 features from the “Large patch16-224” model, and 768 features from the “Base resnet50-224” model were combined to create a new feature set with a total of 2560 features. Then, the set of 2560 features was classified with machine-learning methods (kNN, LDA, and SVM). The confusion matrix obtained as a result of this process is shown in Figure 7. The classification success improved with the merging technique and the introduction of machine-learning methods. The LDA and SVM methods gave the best overall accuracy. The overall accuracy of both was 98.50%. The overall accuracy achieved with the kNN method was 97.01%. The results of this metric are given in Table 5.

**Figure 8:**
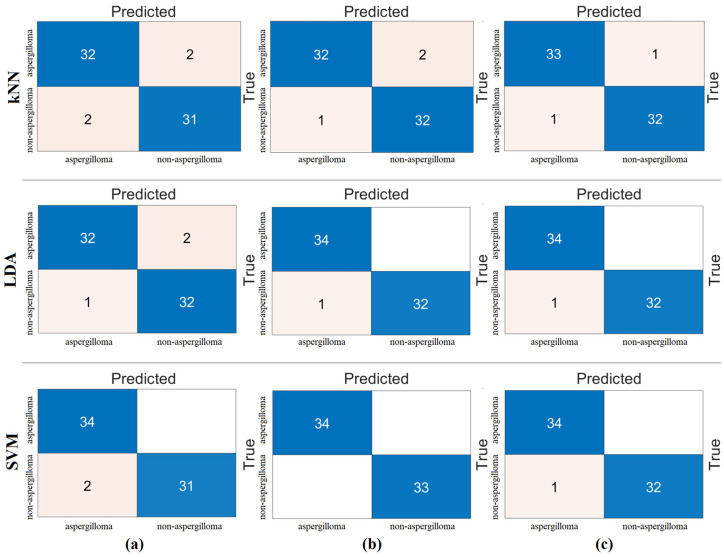
Classification of feature sets obtained by feature selection methods from the combined feature set with machine-learning methods and confusion matrices obtained (training/test rate = 0.8:0.2): (**a**) *Chi*2, (**b**) mRMR, and (**c**) Relief.

**Figure 9:**
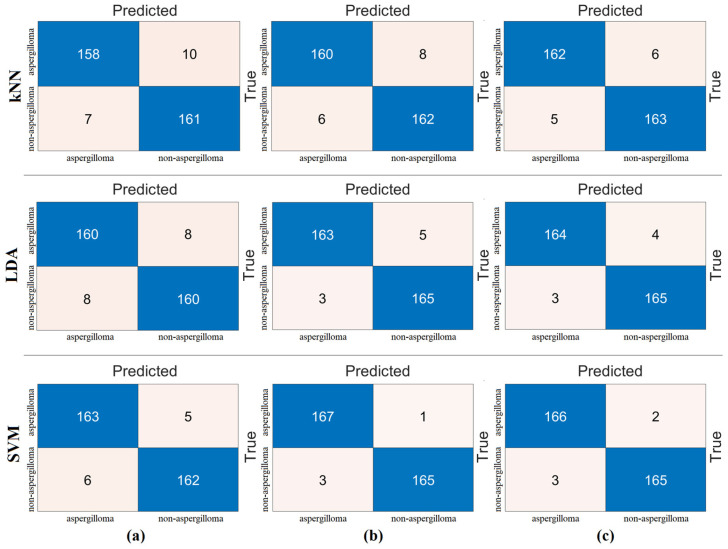
Classification of feature sets obtained by feature selection methods from the combined feature set with machine-learning methods and confusion matrices obtained (cross-validation k = 5 was selected): (**a**) *Chi*2, (**b**) mRMR, and (**c**) Relief.

The third step of the experimental analysis was to highlight efficient features. For this, machine-learning methods (kNN, LDA, and SVM) were used in the classification process. First, the combined feature set (2560 features) was processed with feature selection methods (*Chi*2, mRMR, and Relief). The goal of this step was to observe whether fewer features contribute to the overall performance. The goal was to achieve gains in hardware cost and performance. To achieve this, we identified the best 100 features obtained with feature selection methods and reclassified them with the kNN, LDA, and SVM methods. The reason we settled on the best 100 features is that we also selected the best 200 features or the best 50 features. However, we observed the performance improvement with the best 100 features. Therefore, the feature selection methods processed the combined feature set to identify the best 100 features and then classified them with machine-learning methods. The confusion matrices and analysis results obtained from the analysis of this step are shown in Figure 8 and Table 6, respectively. Figure 8 and Table 6 show that the SVM method produces better analysis results than the LDA and kNN methods. The mRMR method gave the best performance, and 100 features obtained from mRMR were classified by the SVM method. As a result of the classification, the overall accuracy was 100%. This step was also analyzed with cross-validation (k = 5 was chosen). The confusion matrices obtained from the analysis are shown in Figure 9 and the metric results are shown in Table 7. Once more, the SVM method gave better analysis results than the LDA and kNN methods. The mRMR method gave the best performance and 100 features obtained from mRMR were classified with the SVM method. The overall classification accuracy was 98.81%.

**Figure 10:**
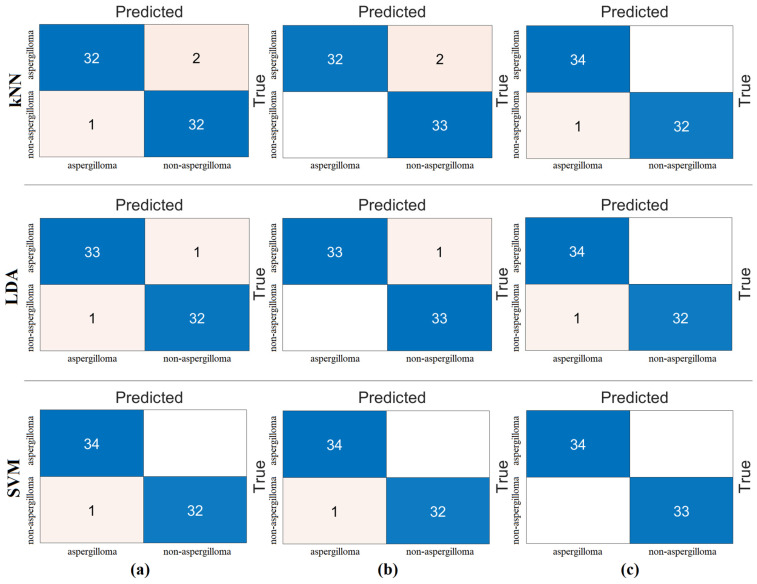
Combining the feature selection algorithms among themselves and reclassifying the new feature set (training/test rate = 0.8:0.2). Confusion matrices obtained as a result of these analyses: (**a**) *Chi*2 and mRMR, (**b**) *Chi*2 and Relief, and (**c**) mRMR and Relief.

In the fourth step, the best features were selected by feature selection methods with a certain limitation (the selection of the best 40 features or the selection of the best 25 features) and the selections of the feature selection methods were combined and classified by machine-learning methods. In the third step, general success was achieved with 100 features. In this stage, we are looking for an answer to the question of whether 100% overall accuracy can be achieved by at least reducing the number of features. In this step, the number of features was determined by trial and error. We achieved success by selecting the best 40 features from each feature selection method. For example, we combined the best 40 features selected by the *Chi*2 method with the best 40 features selected by the mRMR method. The total number of features was 79. The reason why the number of features is not 80 is because there are common feature columns between the two feature selection methods (there is an intersection). In this way, there was a fusion between the *Chi*2 and Relief methods (top 40 features ˅ top 40 features). In the same way, the mRMR and Relief methods were combined (best 40 features ˅ best 40 features). Classification was then performed using machine-learning methods. The best classification success was achieved with the SVM method. The best performance was given by the mRMR and Relief feature set. The mRMR and Relief methods performed best with 79 features and achieved 100% overall accuracy. As a result of this step, we achieved 100% overall accuracy with fewer features. The analysis results of the fourth step are shown in Table 8, and the confusion matrix of the classification analysis performed by combining the feature selection methods is shown in Figure 10. Finally, the proposed hybrid approach for the diagnosis of aspergilloma disease was performed successfully at each step.

**Figure 11:**
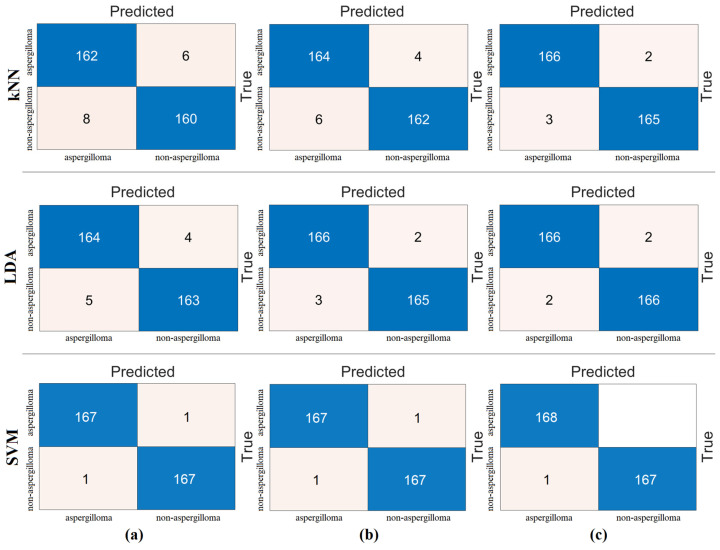
Combining the feature selection algorithms among themselves and reclassifying the new feature set (cross-validation k = 5 was selected). Confusion matrices obtained as a result of these analyses: (**a**) *Chi*2 and mRMR, (**b**) *Chi*2 and Relief, and (**c**) mRMR and Relief.

The fifth step was carried out to validate the analyses of the fourth step or the proposed approach. In order to obtain a more reliable and objective distribution of the data in the analyses, the cross-validation technique was used. Here, the feature sets were processed by cross-validation (k = 5 was chosen) and reclassified by machine-learning methods. The SVM method showed the best performance compared to the other methods (kNN and LDA). The results of the confusion matrices obtained after classification are shown in Figure 11. The metric results of the confusion matrices are also shown in Table 9. As a result of cross-validation, it was observed that the mRMR and Relief feature set gave the best performance. In this step, an overall accuracy of 99.70% was achieved. The combination of the other two feature sets gave a 99.40% overall accuracy with the SVM method. The last step shows that the proposed approach is a successful model for aspegilloma classification. The fifth step gave results that confirmed the fourth step.

## 4. Discussion

Radiologic imaging is usually used to diagnose aspergilloma. Treatment depends on the patient’s overall health and the severity of symptoms. For aspergilloma, which is usually asymptomatic, observation may be sufficient, but, if the symptoms become complicated or severe, medications, surgery, or endoscopic treatments may be used during the treatment process. Early diagnosis is crucial in order to initiate treatments at the earliest and least severe stage of the disease. Contemporary applications leveraging next-generation technologies are increasingly preferred over traditional approaches. This is supported by the growing recognition of AI’s applications across various fields in recent years. In this study, an AI-based hybrid model is proposed for the early diagnosis of aspergilloma disease.

The proposed hybrid model consists of pre-processing steps, model training, and post-processing steps. Each step performed in the proposed approach contributed to the disease diagnosis. This also had a positive effect on the performance of the ViT models. ViTs offer advantages over CNN models, such as the ability to patch the image and efficiently use attention modules for each patch, better capture contextual information, and better adapt to larger or more complex datasets. Although the limited number of datasets in the analysis of this study is a disadvantage for the proposed approach, we compensated for this disadvantage by using a data augmentation technique in the pre-processing step. Combining the feature sets obtained from the last layer of ViT models and classifying them with machine-learning methods provided a higher level of success than the success obtained from individual ViT models. The fact that our preferred transformer models are of the “base” type minimized the cost in terms of parameters and depth compared to other types. Although the balanced nature of the dataset makes the f-score metric lag behind, the disease sensitivity metrics also confirm the success of the proposed approach. However, in the next step, feature selection methods were used to reduce this cost. Thus, more successful results were obtained with fewer features. We went further and combined the best 40 features with feature selection methods between methods and classified them with the SVM method and achieved 100% overall performance success with a total of 79 features. The analysis used 79 features obtained by combining the top 40 features selected by the mRMR and Relief methods. Here, the SVM method gave more successful results than the machine-learning methods (LDA and kNN) used in the experimental analysis. Another limitation of the experimental analysis of this study was the separation of the dataset into training/test slices. Several techniques are used in the literature to avoid this. The most important and preferred of these techniques is cross-validation to achieve a more balanced distribution of the data. We also used the cross-validation technique to verify the overall accuracy of the proposed approach. The results obtained with the cross-validation technique confirmed the accuracy of the proposed approach. Finally, the SVM method achieved a 99.70% overall accuracy in classifying the data processed with the cross-validation technique. In the cross-validation phase, we retrained the ViT models at each fold, performing feature extraction and selection independently, thereby eliminating the risk of data leakage and increasing the generalizability of the model.

There are limited studies in the literature on the diagnosis of aspergilloma disease with AI. When Table 1 is examined, we see that researchers Tong Liu [6], Wei Wang [7], and L.N. Walti [8] have analyzed this disease with AI. In Wei Wang’s study [7], the use of big data images contributed to the training of their proposed CNN model. However, the studies in Table 1 used classical approaches (machine-learning methods, and deep-learning models/CNNs) in their analysis. This also limited their overall success rate. Our proposed approach can achieve similar results on different datasets. Although the accuracy of the model is quite high, it is designed in a robust and reliable way in terms of the generalization capability and implementation. We also trained the original dataset of this study with the other models used in Table 1 (DenseNet-121 and DenseNet-201). Our goal was to check the validity of our dataset with the models used in other studies. We used the parameters preferred for the models in our study also for the models in Table 1. The results of this analysis are presented in Table 10. Examining Table 10, a 77.61% overall accuracy success was obtained by training our dataset with the DenseNet-121 model and an 83.58% overall accuracy success was obtained by training with the DenseNet-201 model. The DenseNet-121 model is the main model used in the IPA-NET model. Wei Wang et al. [7] analyzed the IPA-NET model by improving the DenseNet-121 model (by adding dense layers and fully connected layers). The reason for the high success of Wei Wang et al. [7] here is that they improved the model they proposed.

**Figure 12:**
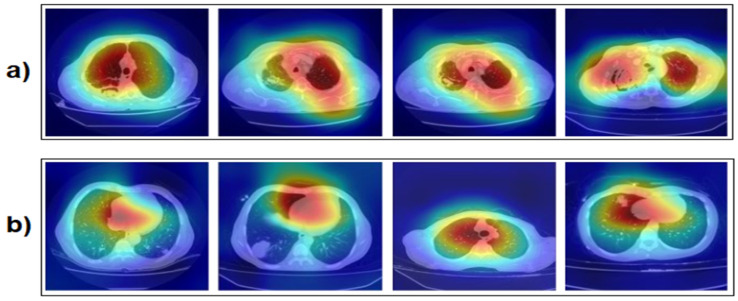
Sample image set obtained by applying the Grad-CAM technique to the dataset: (**a**) aspergilloma and (**b**) non-aspergilloma.

The Gradient-weighted Class Activation Mapping (Grad-CAM) technique was applied to our dataset and retrained with our proposed approach by creating heat maps that highlight areas on the image that affect the classification result. The subset of images processed with the Grad-CAM technique is shown in Figure 12. Each step of our proposed approach was implemented literally. We achieved the best performance by obtaining 64 feature columns with the *Chi*2 and Relief feature selection methods. By classifying the 64 feature columns with an SVM (cross-validation k = 5 was chosen), an overall accuracy of 99.40% was achieved.

The Grad-CAM technique provides more transparency into the model’s decision-making process, making its decisions more understandable by visualizing which regions the model considers more. In this way, the visual features behind the model’s correct classification decisions become easier to understand. The visualizations obtained with Grad-CAM increase its usability in clinical decision support systems. Especially in healthcare, being able to understand not only the accuracy of the model, but also why that decision was made, builds confidence in the diagnostic process of doctors. As a result, the method proposed in this study has provided an important step forward in the early diagnosis of aspergilloma disease, both in terms of accuracy and explainability.

In addition, we analyzed the instances misclassified by our proposed Grad-CAM-based approach. It was observed that misclassifications with the Grad-CAM technique were mainly due to examples with similar visual characteristics. Some of the images belonging to aspergilloma and non-aspergilloma classes had similar textural or stylistic features, which made the model’s decisions difficult. Such cases are one of the factors that negatively affect the performance of the model and we believe that these errors can be reduced in the future with larger datasets or different model configurations.

## 5. Conclusions

Aspergilloma is a disease caused by a fungus called *Aspergillus*, which is usually found in the lung area of the body. The goal of treatment is to relieve symptoms, control infection, and reduce the risk of complications. As with any disease, the early diagnosis of aspergilloma is essential for effective management. This article was conducted to evaluate the contribution of AI approaches in the diagnosis of aspergilloma disease. The proposed approach consists of hybrid processing steps such as image pre-processing steps, ViT model training, feature selection methods, feature fusion techniques, and machine-learning methods. ViT models have been instrumental in obtaining efficient features. In the final step, the mRMR and Relief feature selection and feature fusion methods were used. The classification of the efficient feature set obtained here by the SVM method resulted in an overall accuracy of 100%. The same feature set was processed using a cross-validation technique (k = 5) and classified with an SVM. This analysis resulted in an overall accuracy of 99.70%. This result shows that the proposed hybrid approach contributes to the diagnosis of aspergilloma disease. The contributions of the proposed approach to the literature and diagnosis of the disease are as follows:

- It is critical to select the most appropriate features to make an accurate diagnosis of the disease. In this context, ViT models have proven to be effective.
- It provides objective decisions independent of the interpretation of expert opinions.
- It eliminates differences in interpretation between experts/physicians.
- The proposed approach can make fast and collective decisions in the diagnostic process of many patients.
- Because the proposed approach is designed with next-generation technologies, the opportunities it offers contribute to innovation.

In the next study, the proposed approach will be applied to the detection of various diseases and alternative techniques or methods will be tested to improve the approach. For example, meta-heuristics will be used instead of traditional feature selection methods to identify more efficient features. In the pre-processing phase, model training will be performed on each image using region-of-interest (ROI) techniques. In addition, different ViT models will be evaluated and the models that provide the most efficient results will be analyzed.

## Figures and Tables

**Table 1:** Studies in the AI-based literature on aspergilloma disease.

Article	Model/Method	Accuracy (%)
Tong Liu [6]	DenseNet-201	84.3
Wei Wang [7]	IPA-NET (deep-learning model)	96.8
L.N. Walti [8]	Machine learning	91.7

**Table 2:** Hyperparameters used in machine-learning method.

Machine-Learning Method	Parameter Value/Choice
LDA parameter	
Covariance structure	Full
SVM parameter	
Kernel function	Cubic
Kernel scale	Auto
Box constraint level	1
Multiclass method	One-vs.-One
kNN	
Preset	Cubic kNN
Number of neighbors	10
Distance metric	Minkowski

**Table 3:** Parameters and values of ViTs used in the proposed approach.

Model/Method	Parameter	Preference/Value
ViTs	Loss function	Cross-entropy
	Learning rate	1 × 10^−4^
	Optimization	SGD
	Classifier	Linear
	Epoch	30
	Mini-batch	4
	Training rate: testing rate	0.8:0.2

**Table 4:** Metric results from the confusion matrix of ViT models (%).

ViT Model	Features	Class	*Se*	*Sp*	*Pre*	*F-Scr*	*Acc*
Base patch16-224	768	aspergilloma	100	93.94	94.44	97.14	97.01
		non-aspergilloma	93.94	100	100	96.87	
Large patch16-224	1024	aspergilloma	94.11	90.90	91.42	92.75	92.53
		non-aspergilloma	90.90	94.11	93.75	92.30	
Base resnet50-224	768	aspergilloma	91.17	93.94	93.94	92.53	92.53
		non-aspergilloma	93.94	91.17	91.17	92.53	

**Table 5:** Performance results (%) obtained with machine-learning methods of the combined feature set (2560 features).

Process	Machine Learning	Features	Class	*Se*	*Sp*	*Pre*	*F-Scr*	*Acc*
Feature set combination of ViT models	kNN	2560	aspergilloma	97.05	96.96	97.05	97.05	97.01
			non-aspergilloma	96.96	97.05	96.96	96.96	
	LDA	2560	aspergilloma	100	96.97	97.14	98.55	98.50
			non-aspergilloma	96.97	100	100	98.46	
	SVM	2560	aspergilloma	100	96.97	97.14	98.55	98.50
			non-aspergilloma	96.97	100	100	98.46	

**Table 6:** Performance results obtained from the classification of the top 100 features obtained by feature selection methods with machine-learning methods (training/test rate = 0.8:0.2).

Feature Selection	Machine Learning	Class	*Se*	*Sp*	*Pre*	*F-Scr*	*Acc*
*Chi*2	kNN	aspergilloma	94.12	93.94	94.12	94.12	94.03
		non-aspergilloma	93.94	94.12	93.94	93.94	
	LDA	aspergilloma	94.12	96.97	96.97	95.52	95.52
		non-aspergilloma	96.97	94.12	94.12	95.52	
	SVM	aspergilloma	100	93.94	94.44	97.14	97.01
		non-aspergilloma	93.94	100	100	96.88	
mRMR	kNN	aspergilloma	94.12	96.97	96.97	95.52	95.52
		non-aspergilloma	96.97	94.12	94.12	95.52	
	LDA	aspergilloma	100	96.97	97.14	98.55	98.50
		non-aspergilloma	96.97	100	100	98.46	
	SVM	aspergilloma	100	100	100	100	100
		non-aspergilloma	100	100	100	100	
Relief	kNN	aspergilloma	97.06	96.97	97.06	97.06	97.01
		non-aspergilloma	96.97	97.06	96.97	96.97	
	LDA	aspergilloma	100	96.97	97.14	98.55	98.50
		non-aspergilloma	96.97	100	100	98.46	
	SVM	aspergilloma	100	96.97	97.14	98.55	98.50
		non-aspergilloma	96.97	100	100	98.46	

**Table 7:** Performance results obtained from the classification of the top 100 features obtained by feature selection methods with machine-learning methods (cross-validation k = 5 was selected).

Feature Selection	Machine Learning	Class	*Se*	*Sp*	*Pre*	*F-Scr*	*Acc*
*Chi*2	kNN	aspergilloma	94.05	95.83	95.76	94.89	94.94
		non-aspergilloma	95.83	94.05	94.15	94.99	
	LDA	aspergilloma	95.24	95.24	95.24	95.24	95.24
		non-aspergilloma	95.24	95.24	95.24	95.24	
	SVM	aspergilloma	97.02	96.43	96.45	96.74	96.73
		non-aspergilloma	96.43	97.02	97.01	96.72	
mRMR	kNN	aspergilloma	95.24	96.43	96.39	95.81	95.83
		non-aspergilloma	96.43	95.24	95.29	95.86	
	LDA	aspergilloma	97.02	98.21	98.19	97.60	97.62
		non-aspergilloma	98.21	97.02	97.06	97.63	
	SVM	aspergilloma	99.40	98.21	98.24	98.82	98.81
		non-aspergilloma	98.21	99.40	99.40	98.80	
Relief	kNN	aspergilloma	96.43	97.02	97.01	96.72	96.73
		non-aspergilloma	97.02	96.43	96.45	96.74	
	LDA	aspergilloma	97.62	98.21	98.20	97.91	97.92
		non-aspergilloma	98.21	97.62	97.63	97.92	
	SVM	aspergilloma	98.81	98.21	98.22	98.52	98.51
		non-aspergilloma	98.21	98.81	98.80	98.51	

**Table 8:** Results of combining feature selection algorithms among themselves and reclassifying the new feature set (training/test rate = 0.8:0.2).

Feature Selection	Machine Learning	Features	Class	*Se*	*Sp*	*Pre*	*F-Scr*	*Acc*
*Chi*2 and mRMR	kNN	40 ˅ 40 ≡ 79	aspergilloma	94.12	96.97	96.97	95.52	95.52
			non-aspergilloma	96.97	94.12	94.12	95.52	
	LDA		aspergilloma	97.06	96.97	97.06	97.06	97.01
			non-aspergilloma	96.97	97.06	96.97	96.97	
	SVM		aspergilloma	100	96.97	97.14	98.55	98.50
			non-aspergilloma	96.97	100	100	98.46	
*Chi*2 and Relief	kNN	40 ˅ 40 ≡ 63	aspergilloma	94.12	100	100	96.97	97.01
			non-aspergilloma	100	94.12	94.29	97.06	
	LDA		aspergilloma	97.06	100	100	98.51	98.50
			non-aspergilloma	100	97.06	97.06	98.51	
	SVM		aspergilloma	100	96.97	97.14	98.55	98.50
			non-aspergilloma	96.97	100	100	98.46	
mRMR and Relief	kNN	40 ˅ 40 ≡ 79	aspergilloma	100	96.97	97.14	98.55	98.50
			non-aspergilloma	96.97	100	100	98.46	
	LDA		aspergilloma	100	96.97	97.14	98.55	98.50
			non-aspergilloma	96.97	100	100	98.46	
	SVM		aspergilloma	100	100	100	100	100
			non-aspergilloma	100	100	100	100	

**Table 9:** Results of combining feature selection algorithms among themselves and reclassifying the new feature set (cross-validation k = 5 was selected).

Feature Selection	Machine Learning	Features	Class	*Se*	*Sp*	*Pre*	*F-Scr*	*Acc*
*Chi*2 and mRMR	kNN	40 ˅ 40 ≡ 79	aspergilloma	96.43	95.24	95.29	95.86	95.83
			non-aspergilloma	95.24	96.43	96.39	95.81	
	LDA		aspergilloma	97.62	97.02	97.04	97.33	97.32
			non-aspergilloma	97.02	97.62	97.60	97.31	
	SVM		aspergilloma	99.40	99.40	99.40	99.40	99.40
			non-aspergilloma	99.40	99.40	99.40	99.40	
*Chi*2 and Relief	kNN	40 ˅ 40 ≡ 63	aspergilloma	97.62	96.43	96.47	97.04	97.02
			non-aspergilloma	96.43	97.62	97.59	97.01	
	LDA		aspergilloma	98.81	98.21	98.22	98.52	98.51
			non-aspergilloma	98.21	98.81	98.80	98.51	
	SVM		aspergilloma	99.40	99.40	99.40	99.40	99.40
			non-aspergilloma	99.40	99.40	99.40	99.40	
mRMR and Relief	kNN	40 ˅ 40 ≡ 79	aspergilloma	98.81	98.21	98.22	98.52	98.51
			non-aspergilloma	98.21	98.81	98.80	98.51	
	LDA		aspergilloma	98.81	98.81	98.81	98.81	98.81
			non-aspergilloma	98.81	98.81	98.81	98.81	
	SVM		aspergilloma	100	99.40	99.41	99.70	99.70
			non-aspergilloma	99.40	100	100	99.70	

**Table 10:** Results of applying to the dataset of this study the models used in the following articles.

Article	Model	*Acc* (%)
Tong Liu [6]	DenseNet-201	83.58
Wei Wang [7]	IPA-NET (only DenseNet-121)	77.61
This study	Grad-CAM and our proposed approach	99.40
	Our proposed approach	99.70

## Data Availability

The original contributions presented in the study are included in the article; further inquiries can be directed to the corresponding authors.
